# Supplementary material for: Features of Photosynthesis in Arabidopsis thaliana Plants with Knocked Out Gene of Alpha Carbonic Anhydrase 2
Source: Plants (Basel). 2023 Apr 25;12(9):1763. doi: 10.3390/plants12091763 (PMC10180811; doi:10.3390/plants12091763)
Supplement: Supplementary file 1 [file plants-12-01763-s001.zip › plants-2282556-supplementary.pdf]

Supplementary materials

to manuscript 'Features of photosynthesis in *Arabidopsis thaliana* plants with knocked out gene of alpha carbonic anhydrase 2' by Elena M. Nadeeva, Lyudmila K. Ignatova, Natalia N. Rudenko, Daria V. Vetoshkina, Ilya A. Naydov, Marina A. Kozuleva and Boris N. Ivanov.

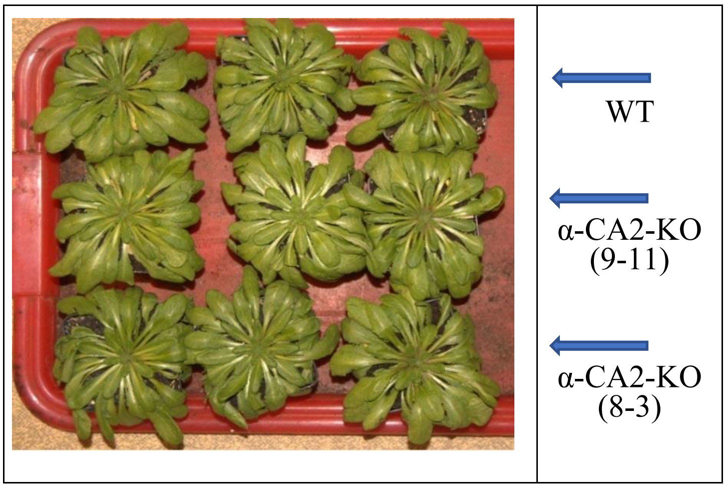

Figure S1. Appearance 55 days old plants of WT and the mutant α-CA2-KO of lines 9-11 and 8-3. Plants were grown in a climatic chamber at a constant temperature of 18-20°C, illumination of 50 µmol quanta m^{-2} s^{-1} (8 h day/16 h night), and CO_2 concentration of 400 ppm. Regardless of the vegetation period, the WT plants and the mutant did not differ in appearance.

Table S1. The weight of the rosette of leaves of one plant WT or the mutant plants α-CA2-KO of lines 9-11 and 8-3. Five plants of each genotype were weighed. Data are presented as the mean from two growing of plants ± the standard error. Significant differences are indicated by *, $p \leq 0.05$.

Plant	Weight, g/one plant rosette
WT	0.966±0.047
α-CA2-KO (9-11)	0.670±0.020*
α-CA2-KO (8-3)	0.675±0.050*

Table S2. Starch content in the leaves of WT plants and α-CA2-KO plants (lines 9-11 and 8-3) in 3 hours of illumination at a light intensity of 400 µmol quanta m^{-2} s^{-1} after 16 hours of dark period. Plants grown at 50 µmol quanta m^{-2} s^{-1} were adapted to light 400 µmol quanta m^{-2} s^{-1} for 5 days at 8 h photoperiod. Data are presented as the mean from two growing of plants, ± the standard error. Significant differences are indicated by *, $p \leq 0.05$.

Plant	Starch content (mg g-1 fresh weight)
WT	12.48 ± 1.04
α-CA2-KO (9-11)	10.19 ± 0.45*
α-CA2-KO (8-3)	8.80 ± 0.85*

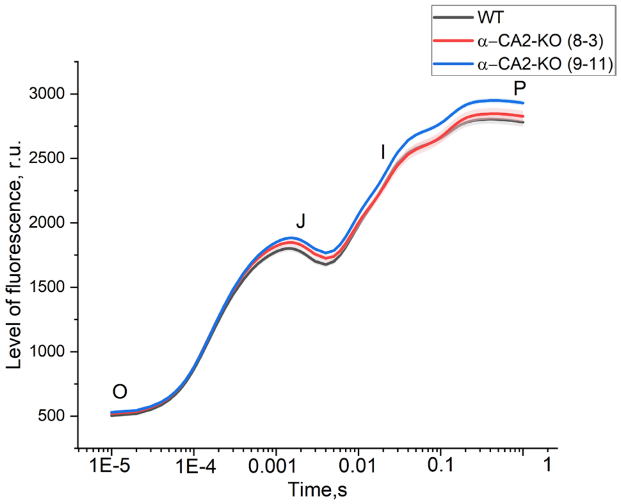

Figure S2. A typical Chlorophyll a polyphasic fluorescence OJIP curve, exhibited by the plants of WT and α-CA2-KO of lines 9-11 and 8-3, grown at low light intensity of 50 μmol quanta m^{-2} s^{-1}. Data are presented as the mean of 15-20 replicates.

A

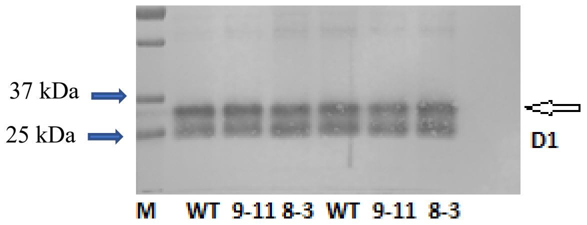

B

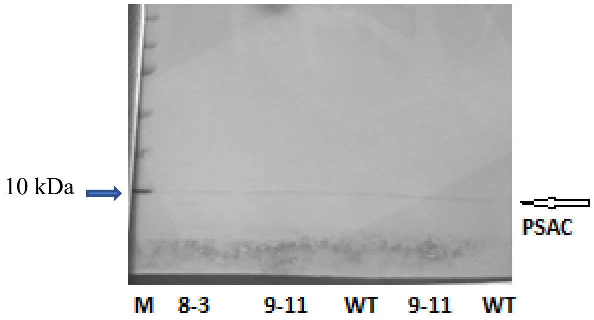

Figure S3. Membranes after Western blot analysis of thylakoid membranes isolated from WT plants and mutants α-CA2-KO of lines 9-11 and 8-3, grown at low light intensity of 50 μmol quanta m^{-2} s^{-1}. A, D1; B, PsaC. Each well was loaded with thylakoid membranes sample containing 1 μg Chl. Two bands on the membrane (A) could be the cause, that antibody against D1 protein can detect the phosphorylated form of D1 or precursor of mature protein as an alternate band to the main band on a high-resolution gel (official site of www.agrisera.com).
